# Long-Read–Based *de novo* Genome Assembly and Comparative Genomics of the Wheat Leaf Rust Pathogen *Puccinia triticina* Identifies Candidates for Three Avirulence Genes

**DOI:** 10.3389/fgene.2020.00521

**Published:** 2020-06-04

**Authors:** Jing Qin Wu, Chongmei Dong, Long Song, Robert F. Park

**Affiliations:** Plant Breeding Institute, School of Life and Environmental Sciences, Faculty of Science, The University of Sydney, Sydney, NSW, Australia

**Keywords:** wheat leaf rust, avirulence genes, secreted protein, long-read assembly, comparative genomics

## Abstract

Leaf rust, caused by *Puccinia triticina* (*Pt*), is one of the most devastating diseases of wheat, affecting production in nearly all wheat-growing regions worldwide. Despite its economic importance, genomic resources for *Pt* are very limited. In the present study, we have used long-read sequencing (LRS) and the pipeline of FALCON and FALCON-Unzip (v4.1.0) to carry out the first LRS-based *de novo* genome assembly for *Pt*. Using 22.4-Gb data with an average read length of 11.6 kb and average coverage of 150-fold, we generated a genome assembly for Pt104 [strain 104-2,3,(6),(7),11; isolate S423], considered to be the founding isolate of a clonal lineage of *Pt* in Australia. The Pt104 genome contains 162 contigs with a total length of 140.5 Mb and N_50_ of 2 Mb, with the associated haplotigs providing haplotype information for 91% of the genome. This represents the best quality of *Pt* genome assembly to date, which reduces the contig number by 91-fold and improves the N_50_ by 4-fold as compared to the previous *Pt* race1 assembly. An annotation pipeline that combined multiple lines of evidence including the transcriptome assemblies derived from RNA-Seq, previously identified expressed sequence tags and *Pt* race 1 protein sequences predicted 29,043 genes for Pt104 genome. Based on the presence of a signal peptide, no transmembrane segment, and no target location to mitochondria, 2,178 genes were identified as secreted proteins (SPs). Whole-genome sequencing (Illumina paired-end) was performed for Pt104 and six additional strains with differential virulence profile on the wheat leaf rust resistance genes *Lr26*, *Lr2a*, and *Lr3ka*. To identify candidates for the corresponding avirulence genes *AvrLr26*, *AvrLr2a*, and *AvrLr3ka*, genetic variation within each strain was first identified by mapping to the Pt104 genome. Variants within predicted SP genes between the strains were then correlated to the virulence profiles, identifying 38, 31, and 37 candidates for *AvrLr26*, *AvrLr2a*, and *AvrLr3ka*, respectively. The identification of these candidate genes lays a good foundation for future studies on isolating these avirulence genes, investigating the molecular mechanisms underlying host–pathogen interactions, and the development of new diagnostic tools for pathogen monitoring.

## Introduction

Leaf rust, caused by *Puccinia triticina* (*Pt*), is one of the most devastating diseases of wheat, affecting production in nearly all wheat-growing regions worldwide. A recent global survey of the impact of pests and pathogens in wheat rated leaf rust as the most damaging, causing losses of approximately 3.25% globally (Savary et al., 2019). To control rust diseases, the most effective and environmentally friendly approach is to grow wheat with resistance (R) genes (Aktar-Uz-Zaman et al., 2017). The proteins encoded by R genes in wheat can recognize effectors encoded by avirulence (Avr) genes in rust pathogens, and upon recognition, plant defense responses known as effector-triggered immunity (ETI) are initiated (Chen et al., 2017). Compared to pathogen-associated molecular pattern–triggered immunity (Jones and Dangl, 2006), ETI is more rapid and robust and is frequently associated with localized cell death known as hypersensitive response. The specific recognition phenomenon between host and pathogen during ETI was first described by Flor (1971) as the gene-for-gene hypothesis. However, host recognition and the ETI response can be evaded by pathogens through the modification of Avr genes (e.g., mutation and deletion), driving host–pathogen coevolution. To date, more than 79 leaf rust (*Lr*) resistance genes have been cataloged in wheat (Mcintosh et al., 2017), many of which including *Lr9*, *Lr14a*, *Lr16*, *Lr17a*, *Lr24*, *Lr26*, and *Lr39* have been overcome by newly detected *Pt* races (Huerta-Espino et al., 2011). The identification of Avr genes and in-depth understanding of host–pathogen interactions are fundamental in developing strategies for durable resistance in wheat and the sustainable control of rust diseases.

The inability to grow obligate biotrophs such as rust fungi readily *in vitro* has hampered biological and genetic studies of these organisms. Next-generation sequencing technology, however, has greatly extended our understanding of rust fungal biology, as demonstrated by the generation of more than 20 rust genomes from 12 rust fungal species (Chen et al., 2019; Lorrain et al., 2019). Following the initial sequencing and assembly of the three rust fungi causing major diseases of wheat, *viz. Puccinia graminis* f. sp. *tritici* (*Pgt*), *Puccinia striiformis* f. sp. *tritici* (*Pst*), and *Pt* (Cuomo et al., 2016), different isolates of these species were also sequenced and assembled with diverse sequencing strategies mostly based on short-read sequencing, for example, *Pgt* race 21-0 (Upadhyaya et al., 2014), *Pst* race 67S64 and 46S119 (Kiran et al., 2017), and *Pt* race 77 and 176 (Kiran et al., 2016). While revealing that rust genomes are characterized by high levels of heterozygosity, a high proportion of repeat elements (as high as >50%), and large numbers of genes (14,000–28,000 per genome) (Cuomo et al., 2016; Lorrain et al., 2019), most rust assemblies published to date are highly fragmented, largely due to the technical limitation of short-read sequencing and the repetitive nature of rust genomes (Aime et al., 2017). To overcome these limitations, long-read sequencing (LRS) has recently been used for *de novo* genome assemblies of *Pst* (Pst104E) and *Puccinia coronata* f. sp. *avenae* (*Pca*), which has generated high-quality genomes with significantly improved contiguity (Miller et al., 2018; Schwessinger et al., 2018). However, a high-quality genome based on LRS is still lacking for *Pt* despite its fundamental importance in comparative genomic studies.

With increasing genome resources becoming available, more and more resequencing studies of wheat rust fungi have been undertaken, enabling comparative genomics for effector mining. For example, comparative studies of five *Pgt* isolates (Upadhyaya et al., 2014), 10 *Pst* isolates (Cantu et al., 2013; Zheng et al., 2013), and 20 Australian *Pt* isolates (Wu et al., 2017) have identified a panel of promising effector candidates for functional validation. Recently, two comparative studies on *Pgt*, one using an isolate of *Pgt* (Pgt279) and a *Sr50* virulent derivative (Pgt632) and the other using ethylmethane sulfonate (EMS)–induced mutant strains, successfully identified *AvrSr50* and *AvrSr35*, respectively, which are the first Avr genes biologically validated and characterized in a wheat attacking rust (Chen et al., 2017; Salcedo et al., 2017). As compared to studies of *Pgt* and *Pst*, comparative studies of *Pt* to identify candidate Avr genes are limited, and our previous study identifying candidates for *AvrLr20* is the only comparative study based on whole-genome sequencing of *Pt* (Wu et al., 2017).

*Puccinia triticina* is not known to undergo sexual recombination in Australia, as the alternative host *Thalictrum* is rare or absent (Park et al., 1995). Pathotype 104-2,3,(6),(7),11 (hereafter referred to as Pt104) was first detected in 1984 and considered to be of exotic origin (Park et al., 1995). It is regarded as the founding isolate of a clonal lineage of putative mutational derivatives that dominated *Pt* populations in all mainland states from 1989 to 2010 (Park et al., 1995, 2000; Park, unpublished data). Following the detection of this founding isolate, a panel of variant pathotypes presumably derived from it through simple step mutation was detected. One of the derivative pathotypes, 104-1,2,3,(6),(7),9,11, carried added virulence for the resistance gene *Lr26* and rendered two cultivars possessing *Lr2*6 susceptible (Park et al., 2000). Within this lineage, isolates with virulences for *Lr2a* and *Lr3ka* were also detected. While *Lr26* has been used widely in many winter and spring wheats and has had a major impact on global wheat production, genes *Lr3ka* and *Lr2a* have been utilized less commonly but have been important when deployed in combination with other resistance genes to achieve multiple gene resistances (Mcintosh et al., 1995).

In the present study, LRS-based *de novo* genome assembly of the founding isolate Pt104 was carried out, generating the best-quality *Pt* genome assembly to date in terms of contiguity and completeness. Transcript-based annotation identified 29,043 genes in the Pt104 genome, of which 2,178 genes were further predicted as encoding secreted proteins (SPs). Six additional isolates presumed to be mutant derivatives of Pt104, along with Pt104, were subjected to Illumina sequencing, and the resequencing data were mapped to the Pt104 assembly to examine genetic variations that may account for the virulence of derivative pathotypes for wheat resistance genes *Lr26*, *Lr2a*, and *Lr3ka*. This approach successfully identified 38, 31, and 37 candidates for *AvrLr26*, *AvrLr2a*, and *AvrLr3ka*, respectively. This study not only provides important new resources for comparative studies of *Pt* in Australia and beyond, but also demonstrates a practical framework of using field-evolved mutational derivatives for Avr gene identification.

## Results

### Long-Read–Based *de novo* Genome Assembly of Pt104

**FIGURE 1:**
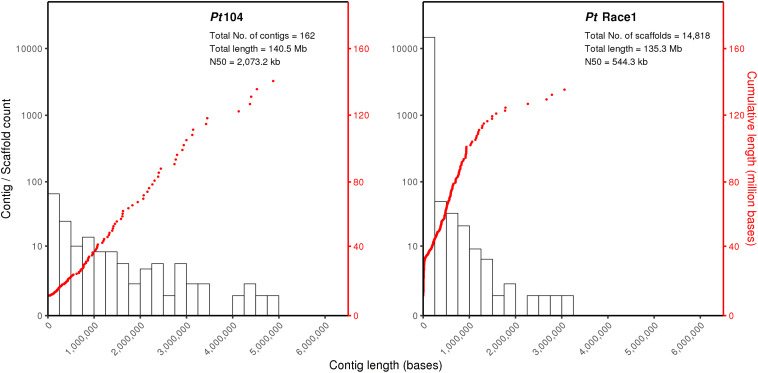
The Pt104 genome assembly with significantly reduced total contig number and improved N_50_ as compared to Pt race1 genome. The log10 counts of contigs within each size bin are shown by histograms with the left *y*-axis. Each dot represents a single contig of a given size corresponding to the *x*-axis. The cumulative sizes of contig lengths sorted from small to large are shown by the dots with the right *y*-axis. The number of contigs or scaffolds, total assembly size, and N_50_ of the assembly are also shown within each plot.

For isolate Pt104, the founding “parental” isolate, LRS data were obtained using three SMRT cells from the PacBio Sequel system. A total of 22.4-Gb data with average read length of 11.6 kb and average coverage of 150-fold were used to generate a *de novo* genome assembly for Pt104 using Falcon and Falcon-Unzip pipeline. After manual curation, the Pt104 genome contained 162 contigs with a total length of 140.5 Mb and N_50_ of 2 Mb (Table 1), with the associated haplotigs providing additional haplotype information for 91% of the genome (Supplementary Table S1). As compared to the previously published *Pt* race1 assembly, our genome substantially improved contiguity as demonstrated by the greatly reduced number of contigs (91-fold; from >14,000 to <200) and the increase in N_50_ statistics (4-fold; contig N_50_ 2,073 kb vs. Scaffold N_50_ 544 kb) (Cuomo et al., 2016; Figure 1 and Table 1). Blastn searches against the NCBI nucleotide reference database showed that none of the contigs had non-eukaryotic sequences as best BLAST hits at any given position.

**TABLE 1:** Pt104 assembly statistics and completeness evaluation.

Assembly statistics	Pt104 genome assembly	*Pt* BBBD race1
Total no. of contigs	162	14,818
No. of contigs with ≥50,000 bp	158	215
Total length (Mb)	140.5	135.3
Total length when ≥50,000 bp	140.3	103.0
Largest contig (Mb)	4.9	3.1
GC (%)	46.7	46.7
N_50_ (kb)	2,073.2	544.3
Complete BUSCOs (%)	92.2	92.6
Complete and single-copy BUSCOs (%)	80.2	89.6
Complete and duplicated BUSCOs (%)	12.0	3.0
Fragmented BUSCOs (%)	3.7	4.3
Missing BUSCOs (%)	4.1/2.6*	3.1

* When the associated haplotigs of Pt104 were combined, the percentage of the missing BUSCO genes of Pt104 assembly was 2.6%.

The completeness of the Pt104 genome assembly was assessed using BUSCO analysis, based on highly conserved fungal genes (basidiomycota_odb9) comprising 1,335 basidiomycete conserved orthologs, which revealed that 92.2% of the BUSCO genes were present as complete sequences (Table 1). The fragmented and missing BUSCO genes were 3.7 and 4.1%, respectively. When the associated haplotigs were combined, the percentage of the missing BUSCO genes was as low at 2.6% (3.1% in *Pt* race1).

The repeat content in the Pt104 genome assembly was evaluated using both *de novo* predicted repeats and fungal elements from RepBase (Bao et al., 2015). The total interspersed repeats of the Pt104 assembly covered 58.4% of the genome (Table 2). Despite the presence of unclassified repeats, the most prevalent repetitive elements were long terminal repeats (>16%).

**TABLE 2:** The repeat contents identified in the Pt104 genome assembly.

Interspersed repeats (%)	Pt104 genome assembly
Long interspersed nuclear elements (LINES)	0.67
Long terminal repeats (LTR) elements	16.86
DNA elements	5.29
Unclassified	35.57
Total interspersed repeats	58.39
**Non-element repeats (%)**	
Simple repeats	1.05
Low complexity	0.07

### Gene Prediction and Functional Annotation

**FIGURE 2:**
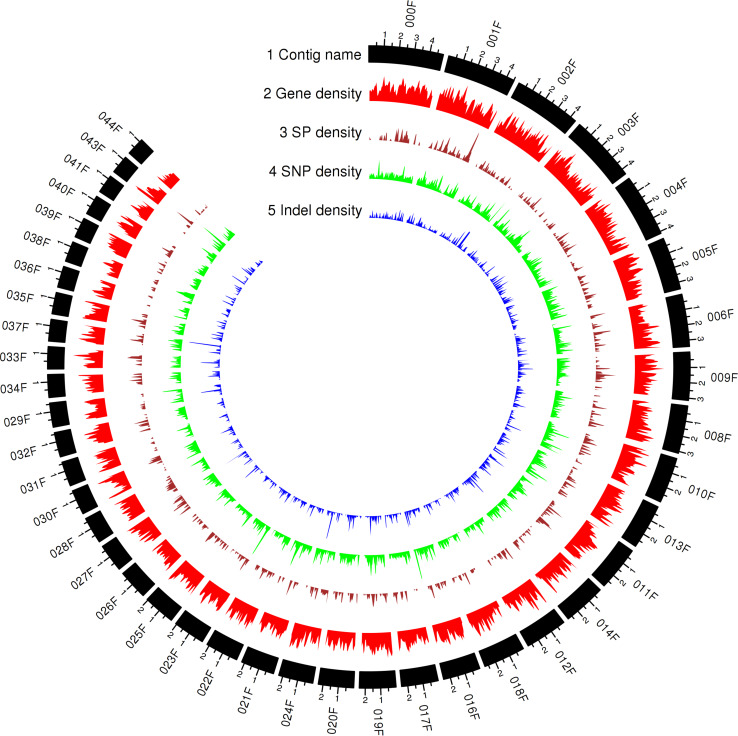
Genomic landscape of predicted gene and secreted protein in Pt104 and genetic variations of the *Pt* isolates represented by the Circos plot of the top 42 contigs ranked by contig length (71% of the Pt104 genome). Tracks from outside to inside are as follows: (1) contig name; (2–5) density of gene; SP, secreted protein SP; SNP, single-nucleotide polymorphism; InDel, insertion or deletion; in non-overlapping 100-kb windows. Each major tick on the contig track is for 1-Mb length.

To capture all genes expressed *in planta*, RNA sequencing data for total RNA extracted from wheat leaves 3, 5, and 7 days after inoculation with Pt104 were obtained. After aligning to the Pt104 genome, fungal specific reads were selected for Trinity to generate both *de novo* and genome-guided transcriptome assemblies (Haas et al., 2013). These mRNA assemblies and the previously reported expressed sequence tags (ESTs) from various stages of the *Pt* life cycle (Xu et al., 2011) as transcript evidence and *Pt* race1 protein sequences as protein evidence were put into the Funannotate v0.7.2 pipeline for gene prediction. This comprehensive approach led to the annotation of 29,043 genes for the Pt104 assembly (Figure 2, Table 3, and Supplementary Table S2).

**TABLE 3:** Gene prediction and functional annotation for the Pt104 assembly.

	Pt104 genome
**Gene prediction**	
Total number of genes	29,043
Mean gene length (bp)	1,378
genome % covered by genes	28.5
Total number of proteins	28,008
**Secretome prediction**	
Secreted proteins	2,178
**Functional annotation**	
CAZy enzymes total number	420
CAZy enzymes GH^a^ number	216
CAZy SP	87
CAZy GH^a^ SP	51
Proteases total number	290
A^b^	21
C^b^	69
M^b^	68
S^b^	98
T^b^	26
I^b^	8
Protease SP	45
A01A	9
S	23
S08A^c^	9
S10^c^	6
C	5
M	6
I51	2

*^*a*^GH, glycoside hydrolase. ^*b*^5 classes of peptidases including S, serine; C, cysteine; M, metallo; T, threonine; A, aspartic proteases as well as one protease inhibitors class (I). ^*c*^S08A contains the serine endopeptidase subtilisin and its homologs and S10 contains only carboxypeptidases.*

**FIGURE 3:**
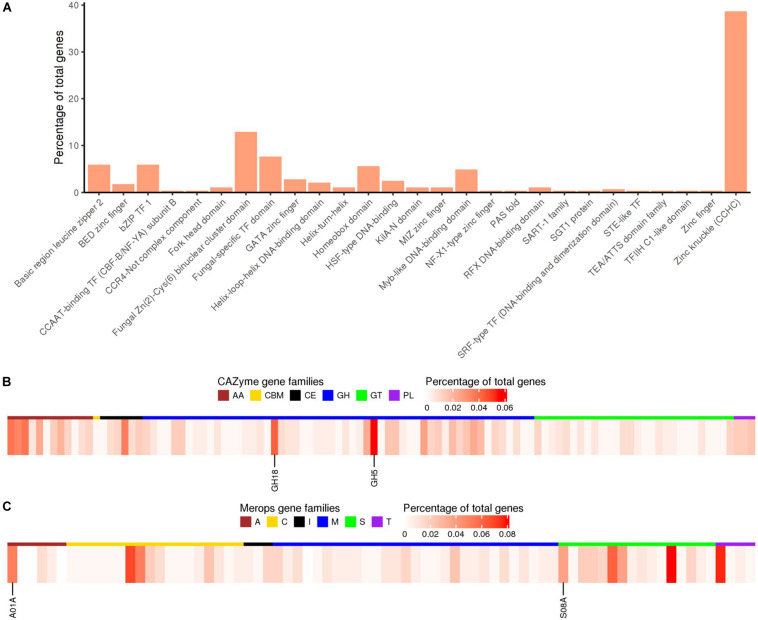
Functional annotation of transcription factors, CAZymes, and Merops proteases for the Pt104 genome. **(A)** Percentages of genes predicted to encode proteins of transcription factor families based on InterProScan annotation. **(B)** Heatmap showing percentages of genes annotated as members of CAZyme families including: AA, auxiliary activities; CBM, carbohydrate-binding modules; CE, carbohydrate esterases; GH, glycoside hydrolases; GTs, glycosyltransferases; PL, polysaccharide lyases. Expanded families GH5 and GH18 are indicated. **(C)** Heatmap showing the percentages of genes annotated as members of the Merops families including: A, aspartic acid; C, cysteine; M, metallo protease; S, serine protease; T, threonine protease; I, peptidase inhibitors. Expanded families A01A and S08A are indicated.

As compared to the previous study on *Pt* race1 focusing largely on core protein comparisons between the three rust pathogens of wheat (Cuomo et al., 2016), our study extended the functional annotation using a range of databases including GO (Gene Ontology), PFAM domains (a large collection of protein families with annotations), interproscan (a database of protein families, domains and functional sites), CAZymes (carbohydrate active enzymes), MEROPS (peptidase database), and transcription factor (TF) families for the Pt104 assembly (Figure 3 and Table 3). Gene Ontology enrichment analysis of the annotated genes revealed no significant overrepresentations or underrepresentations, implicating similar abundances of GO terms. Using the CAZymes database, we detected 420 CAZymes in the Pt104 genome, and the most populated subclass of CAZymes was glycoside hydrolase (GH) enzyme (>200 members; Table 3), with GH5 (cellulases/hemicellulase) and GH18 (chitinase) families being the most abundant ones (Figure 3). Using the MEROPS database, 290 proteases were identified belonging to five classes including serine (S), cysteine (C), metallo (M), threonine (T), and aspartic proteases (A), as well as one protease inhibitor class (I51) (Table 3). As for the TF families, the two top ranked families were zinc finger proteins (Figure 3) including the zinc knuckle CCHC class (IPR001878) and fungal Zn(2)-Cys(6) binuclear cluster domain (IPR001138).

### Secretome Prediction

Proteins possessing a signal peptide, lacking a transmembrane segment, and with no target location to mitochondria were predicted as SPs. We predicted 2,178 SPs on the Pt104 assembly (Figure 2, Table 3, and Supplementary Table S3), comprising approximately 8% of the total proteins, in line with the SP percentages (8–9%) reported for the total predicted proteins in *Pt* race1, *Pst*, and *Pca* (Cuomo et al., 2016; Miller et al., 2018; Schwessinger et al., 2018). Of the 2,178 predicted SPs, 1,530 SP genes had detectable expression levels by *Pt* RNA-sequencing analysis, which was used for Avr gene mining in the subsequent investigation. Of the total CAZymes members, approximately 20% were predicted as SPs and more than 50% of these CAZyme SPs belonged to the GH subclass (Table 3). For the total proteases identified, 15.5% were predicted as SPs. Of these protease SPs, aspartic proteases A01A family and serine peptidases families (S08A of subtilisin-like serine proteases and S10 of carboxypeptidases) were the major types expanded in the Pt104 assembly (Figure 3 and Table 3). All detected protease inhibitors belonged to the I51 family (an inhibitor of serine carboxypeptidase Y that inhibits various kinases), and 25% were predicted as SPs.

### The Mapping of Whole-Genome Sequencing Data of Seven *Pt* Isolates

The Pt104 assembly was used as the reference genome for the mapping of the resequencing data to examine genetic variations that could account for added virulence for resistance genes *Lr26*, *Lr2a*, and *Lr3ka* in the putative derivative mutants of Pt104. Whole-genome sequencing data as 150 base-paired reads from an Illumina HiSeqX platform were generated for the founding isolate Pt104 and six additional strains. Of the six additional isolates, two (S459 and S477) had the same virulence/avirulence as Pt104 but were collected from the field in subsequent years (1988 and 1991, respectively), and four were presumed to be simple mutational derivatives of Pt104, *viz.* S472 with added virulence on *Lr3ka*, S521 with added virulence on *Lr26* and *Lr20*, S474 with added virulence on *Lr2a* and *Lr20*, and S467 with added virulence on *Lr20*. Genomic DNA was extracted from urediniospores of these seven *Pt* isolates, each established from single pustules and characterized for purity and pathogenicity using standard and additional differential wheat lines. Overall, 65 million to 82 million paired-end reads per sample (Table 4) were obtained after quality trimming, which were mapped to the Pt104 genome. The average aligned read depth was 60.6-fold, and the minimum and maximum depths were 53.6 and 72.8-fold, respectively (Table 4). The average mapping rate of these isolates was 90.1%, which covered between 99.2 and 99.4% of the Pt104 reference genome bases.

**TABLE 4:** Mapping information for the seven *Pt* isolates.

Isolate	Total reads (quality trimmed)	Reads mapped to reference	Percentage mapped reads	Average coverage fold	Percentage coverage of reference
S423	81,880,910	76,525,128	93.5	72.8	99.4
S459	64,885,508	60,248,508	92.9	56.9	99.3
S467	71,203,482	66,285,859	93.1	62	99.4
S472	74,627,128	69,765,844	93.5	65.1	99.2
S474	66,019,622	57,035,893	86.4	53.6	99.3
S477	65,750,836	59,783,590	90.9	56	99.3
S521	75,056,408	62,273,511	83.0	58.2	99.3

### Genome-Wide Polymorphism and Phylogenetic Analysis

**FIGURE 4:**
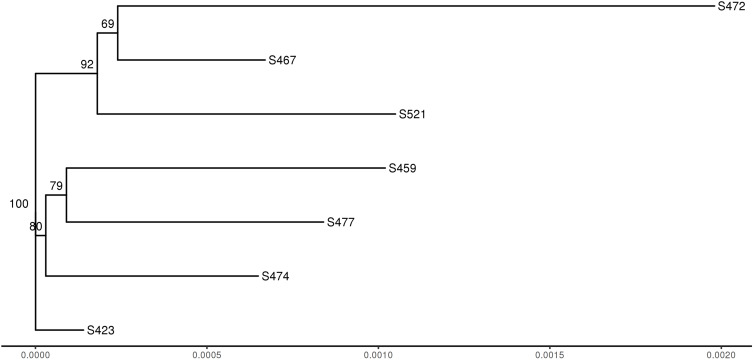
Dendrogram of seven *Pt* strains based on the identified SNPs. The numbers shown on the dendrogram branches are the percentage of bootstrap replicates (1,000) supporting the cluster.

To compare genotypes across the seven strains, genome-wide polymorphisms including single-nucleotide polymorphisms (SNPs) and insertion/deletion (InDel) between individual pathotypes were detected using GATK HaplotypeCaller based on the reads mapped to the Pt104 genome (Figure 2). The average number of total variants identified was 533,799 and the average number of SNP and InDel variants were 454,642 and 79,157, respectively. The average ratio of SNP/InDels was 5.7:1 (Table 5), and the average rates of heterozygous variants (SNP and InDel) and SNPs were 3.5 variants/kb and 3.2 SNPs/kb, respectively. Based on the genome-wide SNPs identified, a phylogenetic tree was inferred (Figure 4), which showed that the six isolates formed two clades along with S423 forming a separate branch. This phylogeny indicated that S423 and the common ancestries of the two clades were closely related. Given that S423 was the first isolate collected and a less developed virulence profile as compared to all of the remaining isolates (Park et al., 1995, 2000), it was plausible to postulate that these six isolates were likely members of a clonal lineage derived from S423 or certain progenitors closely associated with S423 lineage.

**TABLE 5:** Statistics of the genomic variants in the seven *Pt* isolates.

Isolate	Total variants	SNP	InDel	Insertion	Deletion	Heterozygous SNP	Heterozygous InDel
S423	537,561	457,689	79,872	47,613	32,259	453,574	43,838
S459	532,992	454,024	78,968	47,078	31,890	449,609	43,289
S467	534,485	455,196	79,289	47,377	31,912	450,945	43,311
S472	532,048	453,037	79,011	47,189	31,822	447,107	42,999
S474	531,481	452,743	78,738	46,955	31,783	448,199	42,970
S477	532,609	453,671	78,938	47,165	31,773	449,333	43,177
S521	535,415	456,135	79,280	47,246	32,034	451,665	43,406

### Functional Impact of the Genomic Variants

Of the total genomic variants identified, 91,363 (*ca.* 15%) were located within a coding region, covering 16,486 genes in total. The functional impact of these coding variants was further annotated by the Bioconductor package variant Annotation (Obenchain et al., 2014). Amino acid (aa) changes were predicted, and functional consequences were classified into four categories including synonymous (SY), non-synonymous (NSY), frame shift (the variants resulting in sequence length not in a multiple of three), and nonsense (premature stop codons). The average counts in the seven *Pt* strains for each category as aforementioned were 25,648, 45,189, 7,597, and 1,197, respectively (Table 6). Excluding SY mutations, which did not result in an aa change, all remaining categories may have a direct functional impact on *Pt* pathogenicity and hence were included in the subsequent analysis.

**TABLE 6:** Statistics of the functional impacts of the genomic variants in the seven *Pt* isolates.

Isolate	Coding variants	Synonymous variants	Non-synonymous variants	Frameshift variants	Nonsense (premature stop codon) variants
S423	80,039	25,738	45,433	7,648	1,220
S459	79,720	25,690	45,246	7,578	1,206
S467	79,872	25,661	45,388	7,630	1,193
S472	79,211	25,550	44,916	7,562	1,183
S474	79,332	25,604	44,992	7,530	1,206
S477	79,277	25,494	45,021	7,578	1,184
S521	79,966	25,798	45,326	7,652	1,190

### Secretome Genes Associated With Virulence by Differential Genomic Variants

Based on previous studies, we assumed that effectors were most likely encoded by SPs and focused on searching for genomic variants with functional impact located within these genes. For the seven isolates, we identified 2,269 variants with functional impact distributed in 694 SP genes. The variants in these 694 SP genes were inspected manually for read count support and the alignment status, confirming 1,957 variants in 591 SP genes harboring genomic variants with functional impact (Supplementary Table S4). To identify the variations that may account for Avr on *Lr26*, *Lr2a*, and *Lr3ka*, pairwise comparisons were constructed, which included (1) S467 (*Lr26* avirulent) versus S521 (*Lr26* virulent) and S474 (*Lr26* avirulent) versus S521 with both contrasting for *AvrLr26*; (2) S467 (*Lr2a* avirulent) versus S474 (*Lr2a* virulent) and S521 (*Lr2a* avirulent) versus S474 with both contrasting for *AvrLr2a*; and (3) S423 (*Lr3ka* avirulent) versus S472 (*Lr3ka* virulent), S459 (*Lr3ka* avirulent) versus S472, and S477 (*Lr3ka* avirulent) versus S472 with all contrasting for *AvrLr3ka*.

**FIGURE 5:**
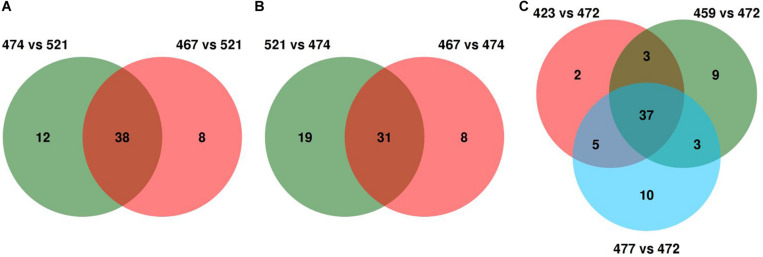
Venn diagrams for the intersection of the candidate genes derived from multiple pair comparisons. **(A–C)** demonstrate the candidates for *AvrLr26* (38), *AvrLr2a* (31), and *AvrLr3ka* (37), respectively.

For each Avr gene, the SP genes with differential variants within each pair were first selected, and those present across pairwise comparisons were considered as potential candidates. For *AvrLr26*, S521 and S467 showed 121 differential variants distributed in 46 SP genes, whereas S521 and S474 showed 98 differential variants distributed in 50 SP genes. Intersecting the two sets led to a common panel of 38 SP genes as the final candidates of *AvrLr26* (Figure 5 and Supplementary Tables S5, S6). Similarly, for *AvrLr2a*, the comparison of S467 versus S474 and S521 versus S474 identified two candidate gene sets with 39 and 50 SP genes, respectively. Intersection of the two sets led to a common panel of 31 SP genes as *AvrLr2a* candidates (Figure 5 and Supplementary Tables S5, S6). For *AvrLr3ka*, the three pair comparisons S423, S459, and S477 versus S472 individually yielded three candidate gene sets comprising 47, 52, and 55 SP genes, respectively. The overlapping of these gene sets identified 37 SP genes as *AvrLr3ka* candidates (Figure 5 and Supplementary Tables S5, S6). As for the variation types of the differential variations leading to the identification of the candidates of *AvrLr26*, *AvrLr2a*, and *AvrLr3ka*, NSY mutations contributed 42% to 50% of the differential variations; frameshifts contributed 8% to 23%; and combinations (e.g., combinations of NSY and frameshift) contributed 32% to 43%; and nonsense was found to contribute only to the identification of *AvrLr3ka* candidates, with a 3% contribution (Supplementary Table S4).

### Biological Functions of Avr Candidate Genes in *Pt*

The Avr candidate genes were further inspected in relation to biological functions and pathogenicity mechanisms. In the aspect of CAZyme activity, two candidates of *AvrLr26* (GN104ID162_008434 from GH5 family and GN104ID162_021096 from GH7 family) and one candidate of *AvrLr2a* (GN104ID162_001475 from GH65 family) belonged to the GH family, and one candidate of *AvrLr3ka* GN104ID162_021071 belonged to the carbohydrate esterase family (CE5) consisting of cutinases, all of which were impacted by NSY mutations (Supplementary Tables S4, S5). For example, the candidate GN104ID162_008434 harbored an NSY mutation at the aa position 369 bearing a change from a charged residue lysine to an uncharged residue glutamine (Supplementary Table S4), which may introduce significant changes in the protein function. The candidates from both GH and CE families have biological functions involved in degrading and loosening plant cell walls, which may enable them to penetrate the protective outer layer of plant tissues (Nakamura et al., 2017). Furthermore, three candidates were predicted to have protease function, including GN104ID162_006831 (metallopeptidase) of *AvrLr26*, GN104ID162_005829 (ubiquitin carboxyl-terminal hydrolase) of *AvrLr2a*, and GN104ID162 _019986 (aspartic peptidase) of *AvrLr3ka*, all of which harbored NSY mutations (Supplementary Tables S4, S5). While several studies have suggested aspartic proteases may act as effectors in rust fungi (Cooper et al., 2016; Jing et al., 2017; Li et al., 2017), a study on *Magnaporthe oryzae* has found that one of the ubiquitin-specific proteases is essential for pathogenicity (Wang et al., 2018). Based on the annotation with InterPro domain as aforementioned (Supplementary Table 5), five of the candidates may be involved in TF-mediated gene regulation, which includes GN104ID162_006801 (IPR001841, zinc finger, RING-type) and GN104ID162_009770 (IPR008917, Skn-1-like TF) of *AvrLr26*, GN104ID162_005718 (IPR001841) and GN104ID162_006814 (IPR001781, zinc finger, LIM type) of *AvrLr2a*, and GN104ID162_006800 (IPR001841) of *AvrLr3ka*. Whereas GN104ID162_006800 had NSY mutations, the remaining four candidates experienced frameshift mutations (Supplementary Table S4).

### Orthologs of the Candidate Avirulence Genes in *Pt* Race1

To inspect our results in the context of previous studies that were based largely on the *Pt* race1 genome, ortholog analyses were carried out for the Pt104 (29,043 genes) and *Pt* race1 genomes (∼15,000 genes), which identified 10,511 entries showing corresponding orthologs between these two genomes (Supplementary Table S7). The orthologs consist of more than 70% of the total genes of *Pt* race1, which reflects a good consistency in gene annotation between the two assemblies. For the *AvrLr26*, *AvrLr2a*, and *AvrLr3ka* candidates identified in the Pt104 genome, 20, 15, and 19 orthologs were found in the *Pt* race1 genome, respectively. Of these orthologs, the *AvrLr2a* candidate GN104ID162_007386, the *AvrLr3ka* candidate GN104ID162_024924, and the *AvrLr26* candidate GN104ID162_020918 had corresponding orthologs of PTTG_07365, PTTG_28070, and PTTG_11943 in the race1 genome, respectively (Supplementary Table S6). In agreement with these findings, these orthologs from *Pt* race1 were also predicted as candidate effectors based on a proteomics study of haustoria isolated from *Pt* race1 (Rampitsch et al., 2015).

## Discussion

The wheat leaf rust fungus *Pt* causes one of the most common diseases of wheat worldwide and is considered to be the most damaging wheat disease globally. Despite its economic significance, genomic resources for this pathogen are relatively limited as compared to the other two wheat rust pathogens *Pgt* and *Pst* (Kiran et al., 2016). In the present study, we generated the first long-read based genome assembly with unprecedented high quality. The assembly is based on the Australian *Pt* pathotype, Pt104, which is the presumed founding isolate of pathotypes that dominated the Australian *Pt* population from 1989 to 2010 (Park et al., 1995, 2000; Park, unpublished data). This LRS-based *Pt* assembly with greatly improved contiguity provides more accurate and richer resources to address central comparative genomics questions such as the identification of Avr genes in *Pt*. In addition to Pt104, we also used Illumina short-read sequencing to generate whole-genome sequencing data for six additional field-collected *Pt* pathotypes presumed to be simple mutational derivatives of Pt104 with stepwise additions of virulence for three resistance genes. The sequencing data of these pathotypes were mapped to the Pt104 genome assembly to identify potential candidates for *AvrLr26*, *AvrLr2a*, and *AvrLr3ka*.

To date, the genomic resources of genome assembly and resequencing that are available for *Pt* remains limited. There are only two studies that document *Pt* genome assemblies and two studies that report whole-genome resequencing of various *Pt* strains including our recent study on *AvrLr20* (Cuomo et al., 2016; Kiran et al., 2016; Wu et al., 2017). For the previous *Pt* assembly, one study used a combination of Sanger sequencing and next-generation pyrosequencing to build draft genome assemblies for races 77 and 106 from India, which were highly fragmented even after scaffolding as exemplified by the small N_50_ of 102.4 kb for race 77 and 20.7 kb for race106 (Kiran et al., 2016). The other study was for the American *Pt* BBD race1, which used various DNA libraries (e.g., fosmid and BAC libraries) and sequencing platforms (e.g., Roche 454 and Sanger sequencing) to build an assembly comprising 14,818 scaffolds with an N_50_ length of 544 kb (Cuomo et al., 2016). Although this assembly has better quality and has been used as a reference genome by a couple of transcriptome and proteome studies as well as our study on *AvrLr20* identification (Song et al., 2011; Bruce et al., 2014; Rampitsch et al., 2015; Wu et al., 2017), the major issue of high fragmentation largely due to the limitation of short-read sequencing and repetitive nature of rust genomes remains to be resolved (Aime et al., 2017). While our LRS-based Pt104 assembly has a genome length close to 135.3 Mb as previously reported for *Pt* race1, our assembly is significantly improved in terms of contiguity and completeness as exemplified by 91-fold reduction in the number of contigs, 4-fold improvement in N_50_ statistics (contigs N_50_ versus scaffolds N_50_; Figure 1 and Table 1), and no missing data represented by Ns (Cuomo et al., 2016). When compared with the recently developed LRS-based rust genomes of *Pst* and *Pca*, with N_50_ length of 1.3 Mb and 268 kb, respectively, our Pt104 assembly has high quality similar to the former, and better than the latter (Miller et al., 2018; Schwessinger et al., 2018). This high-quality Pt104 assembly provides invaluable new resources for comparative genomics and effector identification for the destructive wheat pathogen *Pt*.

Characteristic of the rust fungi genome enriched in repetitive elements, 58.4% of the Pt104 assembly was covered by interspersed repeats (Table 2), higher than the previous report of 51% repeat coverage in *Pt* race1 (Cuomo et al., 2016). Previous studies also noted that the genome expansion in *Pt* was mainly due to repetitive elements and that *Pt* has higher repeat contents than *Pst* (31.5%) and *Pgt* (36.5%) (Fellers et al., 2013; Cuomo et al., 2016). Compared with the new LRS-based *Pst* assembly reporting 54% repeat coverage (Schwessinger et al., 2018), the estimated repeat content of *Pt* remains higher than in *Pst*. While our results confirmed the highly repetitive nature of the *Pt* genome, our LRS-based assembly of Pt104 overcame many of the difficulties caused by such repetition that have led to fragmentation in previously published assemblies of *Pt*.

Our transcript-based annotation of the Pt104 genome identified 29,043 genes (Figure 2, Table 3, and Supplementary Table S2), which is close to the number of genes predicted from *Pt* races 77 and 106 (26,000–27,000) (Kiran et al., 2016), but higher than *Pt* race1 (∼15,000) (Cuomo et al., 2016). The predicted gene number for the Pt104 genome is also in the range of the gene numbers predicted for other rust fungal genomes, such as *Pgt* (22,391) (Upadhyaya et al., 2014), *Pst* (20,000–25,000) (Cantu et al., 2013; Zheng et al., 2013), and *Pca* (26,000–28,801) (Miller et al., 2018). The differences between Pt104 and race1 could be attributed to a number of reasons, such as improved contiguity of the assembly, different gene annotation and filtering methods, and differences between isolates within a species. Nevertheless, keeping a comprehensive set of predicted genes is beneficial for the purpose of Avr gene mining.

Functional annotation of the genes in the Pt104 assembly revealed that a significant portion of the genes annotated in the families of CAZymes, MEROPS, and TF were implicated in the pathogenicity of *Pt*, supporting findings in other rust genome studies (Duplessis et al., 2011; Cooper et al., 2016; Jing et al., 2017; Li et al., 2017). Of the predicted effectors within CAZymes families, 51 (59%) belonged to the GH family (Table 3). Similar to *Pgt* and *Melampsora larici-populina* (*Mlp*) (Duplessis et al., 2011), the GH5 (cellulases/hemicellulase) and GH18 (chitinase) families were most abundant in the Pt104 genome (Figure 3 and Table 3). For the Avr candidates identified, four belonged to the GH families including one from GH5, and these candidates may be related to pathogenicity mechanism involved in degrading and loosening plant cell walls for penetrating host tissues. For the protease effectors, both the aspartic proteases and serine peptidase families were the major types expanded in the Pt104 assembly (Figure 3 and Table 3), which was also seen in *Pgt* and *Mlp* (Duplessis et al., 2011). Notably, 43% of aspartic proteases and 23% of serine proteases were predicted as potential effectors, respectively (Table 3). Previously, serine and aspartic proteases have been suggested to act as effectors in rust fungi (Cooper et al., 2016; Jing et al., 2017; Li et al., 2017), and it has been argued that, in addition to playing a major role in nutrient acquisition, proteases may determine the outcome of plant–pathogen interactions via alternative mechanisms (Lowe et al., 2015). Consistent with these studies, three candidates identified here were predicted as proteases, and the candidate GN104ID162 _019986 of *AvrLr3ka* was the aspartic peptidase, a class that has been implicated in the pathogenicity of rust fungi (Cooper et al., 2016; Jing et al., 2017). For the TF families, two zinc finger protein families were prominent, with the zinc knuckle (CCHC) class containing more than 100 members and fungal Zn (2)-Cys (6) binuclear cluster domain containing more than 30 members (Figure 3). Consistent with previous studies, the CCHC class was also found to be expanded in *Pgt*, *Mlp*, and *Pca* as compared to other fungi (Duplessis et al., 2011; Miller et al., 2018). For the Avr candidates we identified here, four were predicted to belong to zinc finger TF families, which, along with previous studies, highlighted a potentially important role of zinc TFs in rust fungal physiology, possibly involved in the process of effector regulation (Macpherson et al., 2006; Tan and Oliver, 2017).

As for the identification of the candidates for *AvrLr26*, *AvrLr2a*, and *AvrLr3ka*, genome-wide comparisons were made for the seven *Pt* isolates including the founding isolate Pt104 and six presumed mutational derivatives contrasting in virulence profile as described previously. The Illumina sequencing reads of these pathotypes were mapped to the Pt104 genome, and the mapping reads ranged from 83 to 94% with an average rate of 90% (Table 4). Compared to our previously reported 74% to 81% mapping rates of 20 *Pt* isolates to the race1 genome, the current study had approximately 10% improvement in the mapping rate, implying that the Pt104 assembly is a better reference genome for studying Australian *Pt* isolates (Wu et al., 2017). This improvement in mapping rate could be largely attributed to both improved quality of the LRS-based Pt104 assembly and differences between Australian and American isolates within the *Pt* species. Along with the improved mapping rate, we detected an average of 454,642 SNPs per isolate (Table 5), which is approximately 12% higher than the average of 404,690 SNPs identified in our previous study of *AvrLr20*. This improved detection of genomic variants could also be largely related to the improved assembly quality. Based on the genome-wide SNPs identified, a phylogenetic tree was inferred (Figure 4), which was consistent with these isolates being most likely derived from isolates within the S423 lineage or progenitors closely associated with S423 lineage. By including both homozygous and heterozygous polymorphisms, the functional impact of the genomic variants was annotated (Table 6), and the subsequent analysis then focused on the 1,957 variants in 591 SP genes harboring genomic variants with functional impact (Supplementary Table S4). Differential variants derived from the pairwise comparisons set up with contrasting virulence profiles (Supplementary Tables S3, S4) led to the identification of 38, 31, and 37 Avr genes as candidates for *AvrLr26*, *AvrLr2a*, and *AvrLr3ka*, respectively (Figure 5 and Supplementary Table S6). Interestingly, three of the candidate genes had orthologs in *Pt* race1 as aforementioned, which were also predicted as potential effectors in a proteomic study of haustoria isolated from race1 (Rampitsch et al., 2015). This consistency provided further support for our candidate genes at the level of haustorial proteomes. No functional annotation information is available for these three candidates, except that GN104ID162_007386 with ortholog PTTG_07365 was annotated with an InterPro domain of IPR006740, which included a conserved region found in several uncharacterized plant proteins^1^.

Recently, the utility of mutational derivatives in effector mining has been highlighted by two comparative studies that successfully identified two Avr genes in *Pgt*, *AvrSr35* and *AvrSr50* (Chen et al., 2017; Salcedo et al., 2017). Besides the broad criteria of effector prediction including presence of signal peptide, absence of transmembrane segment, and protein localization (Sperschneider et al., 2015), both studies integrated additional criteria to further narrow down the range of the predicted SPs. The *AvrSr50* study focused on the subset of the SP encoding genes (592 haustorial SP) (Chen et al., 2017), whereas the *AvrSr35* study targeted specific CG to TA mutations induced by EMS (Salcedo et al., 2017). In addition to this method, genome-wide association (GWA) mapping has been attempted by several fungal studies to identify pathogenic genetic determinants, including our study on *AvrLr20* (Bartoli and Roux, 2017; Wu et al., 2017). Recently, both GWA and variant comparisons using mutant derivatives have been used in combination to achieve the successful identification of *AvrPm3* effectors (Bourras et al., 2019). This approach demonstrated the potential power of the integrated approach for effector mining in fungal pathogens. Similarly, with more and more sequencing data of *Pt* isolates becoming available, the comparisons of mutant derivatives demonstrated in this study combined with association analysis for the *Pt* population could be attempted in the future.

While whole-genome sequencing techniques have facilitated efficient mining of candidate effectors in rust pathogens, the biological characterization of these candidates remains challenging. Given that *Lr26*, *Lr2a*, and *Lr3ka* have not yet been cloned from wheat, feasible techniques of biological characterization of the corresponding Avr genes include *in planta* expression systems to express the Avr genes in wheat lines containing *Lr26*, *Lr2a*, and *Lr3ka*; RNA interference–based host-induced gene silencing (HIGS) of Avr genes (Lee et al., 2012); and transient expression of the Avr genes in protoplasts (Lu et al., 2016). Although these approaches may allow functional characterization of Avr genes, strong efforts are still needed to improve their accuracy and efficiency. Once high-throughput approaches for functional characterization of candidate Avr genes are established, the identification of Avr genes is expected to accelerate, which will substantially expedite our understanding of the wheat−rust interactions.

In summary, our study has reported the first LRS-based genome assembly of *Pt* with dramatically improved quality, representing the highest-quality and most complete reference genome to date in this species. The in-depth analysis of this genome assembly and resequencing of the derivative pathotypes not only improved our knowledge of genomic variation and gene content in *Pt*, but also led to the successful identification of candidate genes for *AvrLr26*, *AvrLr2a*, and *AvrLr3ka*. The high-quality reference genome and the whole-genome sequencing data of multiple pathotypes provided important new resources for comparative genomics studies of *Pt* in Australia and beyond. In the future, Hi-C sequencing will be obtained to further improve the accuracy of the Pt104 assembly. By mapping Hi-C data to the contigs of the genome assembly, the frequency of contact between pairs of loci can be obtained indicating one-dimensional distance between loci within the genome, which can be exploited to associate and order contigs to large scaffolds (Lajoie et al., 2015; Dudchenko et al., 2017). Integrating the Hi-C approach will yield a more complete assembly at chromosome-scale, which shall further facilitate comparative analysis within and between rust species. With the continuous accumulation of the resources of sequencing data for *Pt*, the approaches of GWA mapping and direct comparisons between derivative strains could be effectively integrated. With the establishment of high-throughput functional characterization of candidate Avr genes, accelerated identification of Avr genes is expected, which will undoubtfully enable a better understanding of the interactions in the *Pt*–wheat pathosystem and expedite the development of durable resistance in wheat and sustainable control of rust disease.

## Materials and Methods

### *Puccinia triticina* Isolates and Plant Inoculation

The *Pt* pathotypes used in this study were identified in nationwide race surveys of pathogenicity in *Pt* in Australia and are curated in the Plant Breeding Institute Rust Collection, The University of Sydney, Australia. To ensure the purity of each isolate for sequencing, a single pustule was selected from a region of low-density infection and propagated on wheat plants of the susceptible variety Morocco prior to DNA preparation. The identity and purity of each isolate were checked by pathogenicity tests with a set of host differentials at each cycle of inoculum increase and also using urediniospores subsampled from those used for DNA extraction. For rust infection, plants were grown at high density (∼25 seeds per 12-cm pot with compost as growth media) to the one leaf stage (∼7 days) in a greenhouse microclimate set at 18°C to 25°C temperature and with natural day light. Plants were inoculated as previously described. For DNA isolation, mature spores were collected, dried, and stored at −80°C.

### DNA Extraction and Genomic DNA Sequencing

DNA was extracted from urediniospores as previously described (Schwessinger and Rathjen, 2017), and PacBio sequencing was performed at the Australian Genome Research Facility Ltd. (Adelaide, Australia). For library preparation, the SMRT cell Template Prep Kit 1.0-SPv3 with BluePippin size-selection with 15- to 20-kb cutoff (PacBio) was used and DNA libraries were sequenced on a PacBio Sequel System with Sequel Sequencing chemistry 2.1. For Pt104, three SMRT cells were used, and each SMRT cell had a 5- to 10-Gb capacity. For Illumina short-read sequencing, TruSeq library of DNA samples for the seven *Pt* races was constructed with a 150-bp paired-end and sequenced on a HiSeqX instrument at Novogene (Hong Kong, China).

### Genome Assembly and Curation

The integrated pipeline of FALCON and FALCON-Unzip (v4.1.0) was used for genome assembly (Chin et al., 2016). Read length cutoffs were computed by FALCON based on the seed coverage and expected genome size. After assembly by Falcon, FALCON-Unzip was used to phase haplotypes and to generate consensus sequences for primary contigs and the associated haplotigs. The generated assembly was subjected to error correction using the final consensus-calling algorithm Quiver implemented in SMRT (v4.0.0), an algorithm for calling highly accurate consensus from PacBio reads using a hidden Markov model exploiting both the base calls and QV metrics to infer the true underlying DNA sequence (Chin et al., 2013). Blastn searches against the NCBI nucleotide reference database were used to check potential non-eukaryotic contamination, and none of the contigs were found to have predominant non-eukaryotic sequences as best BLAST hits at any given position. These assemblies were further curated and polished by removing low quality contigs and reassigning primary contigs without haplotigs showing a significant match with another primary contig. Three manual curation steps were performed using the following criteria for removing low quality contigs or reassigning primary contigs: (1) contigs with extreme low or high coverage (coverage <10- or >2,000-fold) were removed; (2) contigs smaller than 100 kb and >20% of the contigs showing no consensus call marked by Quiver (lowercase) were removed; and (3) primary contigs without haplotigs showing significant match (>85% best match coverage) with another primary contig were reassigned to haplotigs (Roach et al., 2018). To evaluate assembly completeness, the software BUSCO (v3.0) (Simao et al., 2015) was used for comparison with the fungal lineage set of orthologs (basidiomycota_odb9), which consisted of 1,335 conserved orthologs of basidiomycete.

### RNA Isolation and Sequencing

Infected leaves were collected at 3, 5, and 7 days after inoculation with Pt104 and immediately frozen in liquid nitrogen. Samples were ground to a fine powder in liquid nitrogen and total RNA was isolated with the isolate II RNA Mini Kit (Bioline, NSW, Australia). After DNase treatment (Promega, NSW, Australia), RNA was further purified by on-column DNase treatment, and the quality was assessed using the Bioanalyzer 2100. For library preparation, approximately 10 μg of total RNA was processed with the mRNA-Seq Sample Preparation kit (Illumina), which was then sequenced on the Illumina HiSeq2500 platform (125 bp paired-end reads).

### Transcriptome Assembly and Genome Annotation

Quality trimmed RNA-seq reads were first aligned to the Pt104 genome by using the CLC module large gap read mapping (default parameters), and mapped reads were extracted as fungal specific reads. The extracted reads were then used as input to build *de novo* transcriptome assembly using Trinity (v2.1.1) (Haas et al., 2013). Separately, Trinity was also used to build genome-guided transcriptome assembly with the RNA sequencing bam file generated from the CLC. These transcript models along with EST sequences from various life cycle stage of *Pt* (Xu et al., 2011) were then used as transcript evidence, and *Pt* race 1 protein sequences were used as protein evidence for a comprehensive annotation of Pt104 assembly using the Funannotate pipeline (https://github.com/nextgenusfs/funannotate). Funannotate (v0.7.2) is a pipeline specifically developed for fungi genome annotation with an integrated workflow, including repeat identification with RepeatModeler (v1.0.8) and soft masking with RepeatMasker (v4.0.6^2^), alignment of protein evidence to the genomes with TBLASTN and exoneratet (v2.2.0) (Slater and Birney, 2005), alignment of transcript evidence with GMAP (Wu and Watanabe, 2005), *ab initio* gene prediction with AUGUSTUS (v3.2.1) and GeneMark-ET (v4.33) trained by BRAKER1 (Hoff et al., 2016), tRNAs prediction with tRNAscan-SE (v1.3.1) (Lowe and Chan, 2016), generating gene models using EVidenceModeler (v1.1.1) (Haas et al., 2008), and final clean by removing low-quality gene models. After genome annotation, the orthologs between Pt104 and *Pt* race 1 genomes were identified by Proteinortho v5.16 (synteny mode) (Lechner et al., 2011).

### Secretome Prediction and Functional Annotation

Proteins predicted to have a signal peptide with no transmembrane segment and no target location to mitochondria were identified as effector candidates. SignalP v4.1 (Dyrløv Bendtsen et al., 2004), TMHMM v2.0 (Krogh et al., 2001), and TargetP v1.1 (Emanuelsson et al., 2000) were used for the prediction of signal peptide, transmembrane domain, and subcellular location, respectively. Following the gene prediction module as aforementioned, functional annotation to the protein-coding genes was carried out by Funannotate using curated databases including UniProt (Apweiler et al., 2004), Pfam domains (Finn et al., 2014), CAZymes (Yin et al., 2012), MEROPS for proteases (Rawlings et al., 2016), and InterProScan (Jones et al., 2014). The Bioconductor package ComplexHeatmap was used for the plots of the functional annotation (Gu et al., 2016).

### Read Mapping, Variant Calling, and Annotation

After trimming, paired-end Illumina reads of the seven pathotypes were independently mapped to the Pt104 genome using BWA mem v0.7.17 (Li and Durbin, 2009). High-quality alignments (with the mapping quality cutoff of 30) were selected using the SAMTools view command and the generated BAM files were used for SNP calling with GATK v3.8.1. To minimize false positives around InDels, regions around InDels were identified using the GATK RealignerTargetCreator. With the InDel intervals defined, the GATK IndelRealigner was implemented on the BAM alignment files. The re-aligned BAM generated was then used as input to call SNPs and InDels using GATK HaplotypeCaller (Mckenna et al., 2010). Based on the genome-wide SNPs identified, the evolutionary relationships of the strains were inferred using SNPhylo (https://github.com/thlee/SNPhylo) with the performance of 1,000 bootstrap replicates and visualized by Ggtree (Yu et al., 2018). The identified SNPs and InDels were visualized by the R package Circlize (Krzywinski et al., 2009) and annotated with the Bioconductor package variantAnnotation (Obenchain et al., 2014), which predicted and classified the functional impact of the variants into different categories such as SY, NSY, and frame shift. To manually check the variant calls produced by GATK, reads were mapped to the reference genome using bowtie2 v2.2.5 (Langmead and Salzberg, 2012) with parameters “-sensitive-local.” The resulting bam files of read alignments were visualized in IGV for the confirmation of the GATK variant calls of the SP genes.

## Data Availability Statement

The raw datasets generated for this study will be available upon publication in the NCBI BioProject PRJNA607157.

## Author Contributions

JW analyzed the data and wrote the manuscript. CD extracted the high molecular DNA required for PacBio sequencing. LS contributed to data analysis and prepared the figures. RP identified all the pathotypes used and supervised the work. CD, LS, and RP contributed to the manuscript. RP and JW designed the experiment. All authors read and approved the final manuscript.

## Conflict of Interest

The authors declare that the research was conducted in the absence of any commercial or financial relationships that could be construed as a potential conflict of interest.

## Footnotes

1 https://www.ebi.ac.uk/interpro/entry/InterPro/IPR006740/

2 http://www.repeatmasker.org/

## Supplementary Material

TABLE S1 Statistics of the associated haplotigs of Pt104 genome.

TABLE S2 Functional annotation of Pt104 genome.

TABLE S3 Secreted protein genes of Pt104 genome.

TABLE S4 Genomic variants with functional impact in secreted protein genes.

TABLE S5 Biological functions of the Avr candidate genes.

TABLE S6 Candidate genes identified for *AvrLr26*, *AvrLr2a*, and *AvrLr3ka* and the associated orthologs in *Pt* race1.

TABLE S7 Orthologs between Pt104 and *Pt* race1 genomes.
