# Supplementary material for: Long-Read–Based de novo Genome Assembly and Comparative Genomics of the Wheat Leaf Rust Pathogen Puccinia triticina Identifies Candidates for Three Avirulence Genes
Source: Front Genet. 2020 Jun 4;11:521. doi: 10.3389/fgene.2020.00521 (PMC7287177; doi:10.3389/fgene.2020.00521)
Supplement: TABLE S1 — Statistics of the associated haplotigs of Pt104 genome. [file Table_1.DOCX]

**Table 1** **Statistics of the associated haplotigs of Pt104 genome**

| **Assembly statistics** | **Pt104 associated haplotigs** |
| --- | --- |
| Total No. of contigs | 713 |
| No. of contigs with >= 50,000 bp | 304 |
| Total length (Mb) | 128.0 |
| Total length when >= 50,000 bp | 114.2 |
| Largest contig (Mb) | 3.3 |
| GC (%) | 46.7 |
| N50 (kb) | 815.6 |
| Complete BUSCOs (%) | 84 |
| Complete and single-copy BUSCOs (%) | 72.6 |
| Complete and duplicated BUSCOs (%) | 11.4 |
| Fragmented BUSCOs (%) | 4.6 |
| Missing BUSCOs (%) | 11.4 |
